# Antipsychotic drugs and their effects on cognitive function: protocol for a systematic review, pairwise, and network meta-analysis

**DOI:** 10.1186/s13643-023-02213-5

**Published:** 2023-03-24

**Authors:** Lena Feber, Natalie Peter, Johannes Schneider-Thoma, Spyridon Siafis, Irene Bighelli, Wulf-Peter Hansen, Daniel Prates Baldez, Georgia Salanti, Richard S. E. Keefe, Rolf R. Engel, Stefan Leucht

**Affiliations:** 1 Department of Psychiatry and Psychotherapy, Klinikum rechts der Isar, School of Medicine, Technical University of Munich, Munich, Germany; 2 BASTA—Bündnis für psychisch erkrankte Menschen, Munich, Germany; 3 Hospital de Clinicas de Porto Alegre, Universidade Federal Do Rio Grande Do Sul (UFRGS), Porto Alegre, Brazil; 4 Institute of Social and Preventive Medicine (ISPM), University of Bern, Bern, Switzerland; 5 Department of Psychiatry and Behavioral Sciences, Duke University Medical Center, Durham, NC USA; 6 Department of Psychiatry and Psychotherapy, Ludwig Maximilians University, Munich, Germany

**Keywords:** Schizophrenia, Psychosis, Antipsychotics, Cognition, Network meta-analysis

## Abstract

**Background:**

There is evidence that antipsychotic drugs differ in their effect on the cognitive symptoms of schizophrenia. So far, there is no comprehensive systematic review available that would enable providers and patients to make informed choices regarding this important aspect of treatment. With a large number of substances available, conventional pairwise meta-analyses will not be sufficient to inform this choice. To fill this gap, we will conduct a network meta-analysis (NMA), integrating direct and indirect comparisons from randomized controlled trials (RCTs) to rank antipsychotics according to their effect on cognitive functioning.

**Methods:**

In our NMA, we will include RCTs in patients with schizophrenia or schizophrenia-like psychoses comparing one antipsychotic agent with another antipsychotic agent or placebo that measures cognitive function. We will include studies on patients of every age group, in any phase of illness (e.g., acute or stable, first episode or chronic schizophrenia, in- or outpatients) with an intervention time of at least 3 weeks. The primary outcome will be the composite score of cognitive functioning, preferentially measured with the test battery developed by the Measurement and Treatment Research to Improve Cognition in Schizophrenia (MATRICS) initiative. The secondary outcomes include the seven cognitive domains that the composite score is composed of, as well as functioning and quality of life. Study selection and data extraction will be conducted by at least two independent reviewers. We will use the Cochrane Risk of Bias tool 2 to determine the risk of bias in studies, and we will evaluate the confidence in the results using Confidence in Network Meta-Analysis (CINeMA). We will perform NMA using R (package netmeta). We will conduct subgroup and sensitivity analyses to explore the heterogeneity and assess the robustness of our findings.

**Discussion:**

This systematic review and network meta-analysis aims to inform evidence-based antipsychotic treatment choice for cognitive deficits in schizophrenia patients by analyzing existing RCTs on this subject. The results have the potential to support patients’ and physicians’ decision-making processes based on the latest available evidence.

**Systematic review registration:**

PROSPERO CRD42022312483

**Supplementary Information:**

The online version contains supplementary material available at 10.1186/s13643-023-02213-5.

## Background

Schizophrenia is among the 25 leading causes of years lived with disability in the age group 25–49 years, according to the 2019 Global Burden of Disease Study [1]. Besides the well-known positive and negative symptoms, cognitive deficits are another independent core part of schizophrenia. Areas of impairment include memory, attention, processing speed, and executive function, as well as higher cognitive functions such as reasoning and social cognition. These deficits not only affect a large proportion of patients [2, 3]—often, the cognitive symptoms precede the onset of positive symptoms for several years and persist long after the acute positive symptoms have subsided [2, 4]. It has been shown that cognitive dysfunction is a strong, discrete predictor of functional outcomes and quality of life in schizophrenic patients [5]. Apart from negative symptoms, the cognitive deficits present in many patients with schizophrenia pose the main obstacle for keeping up employment and social life alike [6].

While antipsychotic drugs constitute a central part of the treatment of schizophrenia in acute phases and relapse prevention [7–9], we are only beginning to understand which substance works best for which patient and for which facet of their symptoms. Antipsychotics can differ in several aspects, including in their mechanism of action, receptor-binding profile, efficacy, and side effects. While doctors are often able to control the positive symptoms relatively quickly with antipsychotic drugs, negative and cognitive symptoms are often not as straightforward to address. Evidence on the differential effect of individual antipsychotic substances is available but inconclusive. It has been claimed that the newer, traditionally called “second-generation” antipsychotics are superior to older, “first-generation” antipsychotics in terms of cognitive function [10].

There are multiple randomized controlled trials on this issue as well as a few meta-analyses on the comparative effects of antipsychotic drugs on cognition, but none of them incorporates the latest evidence and adheres to the highest quality standards at the same time [10, 11]. Moreover, the only available bigger meta-analysis [11] did not take into account the most recent principles for the assessment of cognitive symptoms in schizophrenia trials as suggested by the Measurement and Treatment Research to Improve Cognition in Schizophrenia (MATRICS) initiative. The MATRICS initiative set out to improve the treatment of cognitive symptoms in schizophrenia and related disorders, identifying the cognitive domains of interest and developing a comprehensive test battery (MATRICS Consensus Cognitive Battery (MCCB)) for use in clinical trials. The MCCB is now considered the international gold standard for the measurement of cognition in this setting [12–14].

## Objective

In summary, high-quality meta-analyses on the effects of antipsychotics on cognition are needed, but not available. To overcome this gap, we will conduct a network meta-analysis examining the effects of different antipsychotic drugs on cognitive function in patients with schizophrenia. Network meta-analysis combines the direct evidence observed in clinical trials with indirect evidence, therefore investigating every antipsychotic in comparison with every other antipsychotic and in consequence producing hierarchies between the antipsychotics according to their effect on overall cognitive function and the specific cognitive domains. In doing so, we aim to summarize the available evidence in an accessible and comprehensible form and thus enable patients and providers to make better, evidence-based treatment choices together.

## Methods/design

This systematic review and network meta-analysis protocol will follow the PRISMA guidelines, extension for network meta-analysis [15]. The PRISMA-P Checklist can be found in Additional file 1. This protocol has been registered with PROSPERO [16]. We will update the report in PROSPERO with any necessary amendments.

### Eligibility criteria

#### Characteristics of studies

We will include randomized controlled trials comparing one antipsychotic agent to at least one other antipsychotic or placebo for the treatment of schizophrenia or schizophrenia-like psychoses. Study arms using combinations of antipsychotics or combinations with other drugs will be excluded. We will include double-blind, single-blind, and open-label studies with an intervention phase of at least 3 weeks. Cluster-randomized trials will be excluded due to unit of analysis problems and to avoid violating the transitivity assumption [17]. In cross-over studies, we will only use the first intervention phase to avoid carry-over effects.

#### Characteristics of participants

We will include studies in which at least 80% of the patient sample has a primary diagnosis of schizophrenia or schizophrenia-like psychosis. We will include trials irrespective of the diagnostic criteria used. Studies in which all patients by inclusion criteria had a comorbid medical or psychiatric illness (including comorbid substance abuse) will be excluded, as these comorbidities or their treatment can have an influence on cognition and could bias the results. There will not be any restrictions in terms of gender, ethnicity, age, phase or severity of illness (first episode, acute episode of chronic illness, stable phase, treatment-resistant, predominant or prominent negative symptoms), or setting (in- and outpatients), but some of these factors will be examined in subgroup and meta-regression analyses.

#### Interventions

We will include a wide range of antipsychotic agents in oral or injectable form with the exception of short-acting injectables, because these are generally only used for emergency treatment. Our choice of included first-generation antipsychotic drugs is based on a systematic survey of schizophrenia experts [18]. The newer antipsychotic drugs are nowadays the most prescribed medication for schizophrenia and are therefore an obvious choice for inclusion. We included all “second generation” drugs currently available in Europe or the USA. Fixed-dose studies will only be included if the doses are within the range of the International Consensus Study on Antipsychotic Dosing [19]. This restriction will not apply to certain participant subgroups (children, elderly, first episode, or treatment-resistant patients) where different dose ranges seem to be more adequate. We will include all flexible-dose studies, as these allow the investigators to titrate to the adequate dose for the individual patient.

### Outcome measures

We will exclusively consider studies that use validated psychometric tests to assess participants’ performance in at least one of the seven domains determined by the MATRICS initiative or that provide an adequate composite score for all domains. These seven domains, identified as particularly important for people with schizophrenia, are speed of processing, attention/vigilance, working memory, verbal learning, visual learning, reasoning and problem solving, and social cognition [20].

As the MATRICS consensus was developed relatively recently (the main publications came out in 2008), and has since also not been adopted in every study on cognition in schizophrenia, we will include tests outside the MCCB, based on their equivalence to the original MCCB tests for the composite score or one of the seven domains. The selection of these tests and the assignment to the respective domain will be made independently by two reviewers and will be supervised by a neuropsychological expert.

If multiple tests for one domain are reported, we will select the most appropriate of them following the principles outlined in this paragraph. We decided to avoid summarizing different tests for the same domain, as this could cause psychometric problems that can affect the validity of the combined scores and their comparability with the results of single test scores [21]. Exceptions will be made for congregate domain scores as defined by MATRICS for the domains speed of processing and working memory, which require multiple tests in the MCCB as well. We will prioritize these over the single MATRICS tests. Other congregate scores per domain or for overall cognition will be judged on a case-by-case basis and will only be considered if they are part of an established neuropsychological test battery.

For determining the most appropriate test within the domain, we will prioritize tests from the final MCCB over tests from the MATRICS beta battery over other tests. In case of multiple MATRICS tests (MCCB or beta battery) for one domain, we will choose the one with the highest intra-class correlation and the highest accordance with the quality criteria used in the development of the MCCB [14]; in case of multiple non-MATRICS tests, we will choose the one with the highest similarity to the MCCB. All decisions regarding the measurement hierarchy will be made independently by two experts in psychometry (RRE, RSEK); in case of disagreement, we will resolve it by discussion.

#### Primary outcome

Our primary outcome will be the composite score for the seven cognitive domains, preferably based on the MCCB [12–14]. Other composite scores of overall cognition reported in the trials will be evaluated on a case-by-case basis.

#### Secondary outcomes

Our secondary outcomes will include measures of one of the seven cognitive domains, as well as rating scales for functioning or quality of life (QoL). MATRICS recommends a selection of co-primary measures to assess functioning but does not include more direct measures. Hence, we will use them only if the suggested tests are not available [12], examples include the Global Assessment of Functioning Scale (GAF), the Social and Occupational Functioning Assessment Scale (SOFAS), or the Personal and Social Performance Scale (PSP). For QoL scales, MATRICS provides no recommendations. In case of multiple tests on the quality of life, we will include quality criteria and how well-known the test is, for our selection. We will follow especially the recommendations of the COSMIN initiative, that aims to improve the selection of health measurement instruments based on their psychometric properties [22]. They propose the following superior domains to evaluate the quality of measurement, if available: reliability, validity, responsiveness, and interpretability [22]. These criteria should help us in our selection process in case of multiple quality of life scales (Table 1).

**Table 1:** MCCB tests and co-primary measurements of functioning

Cognitive domain	Final battery	Beta battery
Speed of processing	Brief Assessment of Cognition in Schizophrenia (BACS): symbol coding subtest Category fluency: animal naming Trail Making Test: part A	Wechsler Adult Intelligence Scale 3rd Ed. (WAIS-III): digit symbol-coding subtest
Attention/vigilance	Continuous Performance Test—Identical Pairs Version (CPT-IP)	3–7 Continuous Performance Test – shortened version
Working memory	Wechsler Memory Scale 3rd Ed. (WMS-III): spatial span subtest Letter-Number-Span test	Brief Assessment of Cognition in Schizophrenia (BACS): digit sequencing subtest Wechsler Adult Intelligence Scale 3rd Ed. (WAIS-III): letter number sequencing subtest Spatial delayed response task
Verbal learning	Hopkins Verbal Learning Test – revised (HVL-R): immediate recall	Neuropsychological Assessment Battery: daily living memory subtest
Visual learning	Brief Visuospatial Memory Test – Revised (BVMT-R)	Neuropsychological Assessment Battery: shape learning subtest
Reasoning and problem solving	Neuropsychological Assessment Battery (NAB): mazes subtest	Wechsler Adult Intelligence Scale 3rd Ed. (WAIS-III): block design subtest Brief Assessment of Cognition in Schizophrenia (BACS): Tower of London subtest
Social cognition	Mayer-Salovey-Caruso Emotional Intelligence Test (MSCEIT): managing emotions branch	Mayer-Salovey-Caruso Emotional Intelligence Test (MSCEIT): perceiving emotions branch
Co-primary measures		
Functioning	Maryland Assessment of Social Competence UCSD Performance-Based Skills Assessment Schizophrenia Cognition Rating Scale Clinical Global Impression of Cognition in Schizophrenia	

#### Search strategy

We have searched the Cochrane Schizophrenia Group Registry of Trials [23] for published and unpublished reports of randomized controlled studies relevant to our research question up to April 27, 2020 (see also Additional file 2) and are planning to conduct an update search. The detailed search strategy for the Cochrane Schizophrenia Group Trials Registry can be found in Additional file 3 [24]. No date or language restrictions will be applied. In addition, we will search the reference lists of previous reviews on the effect of antipsychotic drugs on cognitive function. In case of missing outcome information from included studies, we will try to retrieve it from the corresponding author or responsible drug company.

#### Identification and selection of studies

Two reviewers from our team will independently screen the search results for general inclusion criteria in Citavi [25] and in a second step (LF, NP) for the availability of cognition measurements. Disagreement will be resolved by discussion or consulting a third, experienced reviewer (SL/JST). As the third step, the measurement of cognition is considered in more detail and references with only inappropriate tests will be excluded (e.g., self-reported or interviewer-assessed cognitive impairment, not separately validated modifications of established tests). Disagreement in the evaluation of appropriate measurements will be resolved by discussion with neuropsychological experts (RRE, RSEK).

#### Data extraction

The two reviewers (LF, NP) will extract data from all selected trials in a Microsoft Access database. Our database is specifically developed for studies on schizophrenia and allows a standardized process. The software will automatically detect discrepancies between the two reviewers. When disagreement arises, we will resolve it by discussion and, if needed, by involving a third, senior reviewer. Information on the following points will be extracted:

- General study information (e.g., author name, year, treatment arms)
- Information on methodology (e.g., duration, blinding, diagnostic criteria used)
- Characteristics of the study participants (e.g., subgroup, age, number of men/women, race/ethnicity)
- Characteristics of the used antipsychotics (e.g., doses)
- Outcome measures

#### Measurement of treatment effect

We will use the mean differences for the same scales and standardized mean differences (SMD) for different scales of the same outcome parameter. We will prefer the results obtained with imputation methods to handle missing data over completers’ data and results from mixed models of repeated measurement (MMRM) or multiple imputations over last observation carried forward (LOCF). We will extract standard deviations (SD) as a measure of imprecision, and standard errors (SEs) will be converted to SDs. If both are missing, we will estimate SDs from confidence intervals, *t*-values, or *p*-values as described in the Cochrane Handbook for Systematic Reviews [26]. If none of the options is viable, we will contact the original study authors. In case of no information on SDs, we will use the SD from another study using the same test. If there are many studies using the same test, we will build a weighted mean of the given SDs.

#### Risk of bias assessment

Two reviewers will independently assess the risk of bias using the Cochrane Risk of Bias tool version 2 (RoB 2.0) [27]. We will assess the risk of bias in respect of our primary outcome. In case of disagreement between the two reviewers, we will discuss it with a third, senior reviewer. We will use the RoB-MEN framework to evaluate the risk of bias due to missing evidence in network meta-analysis [28].

### Data analysis

#### Conventional pairwise meta-analyses

We will use a random effects frequentist model for our pairwise meta-analyses.

#### Assessment of heterogeneity

We will measure heterogeneity with tau-squared (the between-study variance). The heterogeneity variance will be assumed common across the various treatment comparisons, and the empirical distributions will be used to characterize the amount of heterogeneity as low, moderate, or high using the first and third quantiles [29, 30]. We will explore the potential reasons for heterogeneity by subgroup and meta-regression analyses.

#### Network meta-analysis

Network meta-analysis combines direct and indirect evidence for all relative treatment effects and can therefore provide estimates with maximum power and increased precision [31]. We will depict all available direct comparisons in network plots for each outcome. We will test the transitivity assumption by investigating if clinical and methodological variables—which can act as effect moderators—are similarly distributed across studies grouped by comparison.

We will perform a statistical evaluation of the assumption of transitivity (often termed consistency) using the design-by-treatment interaction test that evaluates inconsistency from all possible sources in the network jointly [32], as well as by the SIDE test (separating indirect evidence from direct evidence) [33], assessing the agreement of indirect and direct evidence for every possible comparison in the network. In case of evidence of inconsistency or intransitivity, we will investigate possible sources (mistakes in data entry, differences in study characteristics). Small or moderate amounts of inconsistency will be further explored by network meta-regression and subgroup analyses using the potential effect modifiers listed below. We will estimate the probability for each intervention to be ranked at each possible place, given the relative effect sizes as estimated in NMA. We will present the results for all cognition outcomes ranking the interventions by their P-Score [34].

If the requirements for network meta-analysis are not met, we will present only the findings of the pairwise syntheses.

#### Subgroup analysis and meta-regression

We plan to investigate the impact of potential effect modifiers via network meta-regressions or subgroup analyses. We will examine the following characteristics: baseline severity of symptoms, inclusion of acutely ill or stable patients, age, co-medication with anticholinergics, co-medication with benzodiazepines, and dose of antipsychotics. We will examine the confidence intervals of the regression coefficients and compare the heterogeneity and inconsistency of the unadjusted and adjusted (network meta-regression) models to infer about the impact of the effect modifiers. Results will be interpreted with caution, given the observational nature of the examined associations.

#### Sensitivity analyses

We plan to conduct the following sensitivity analyses: excluding (a) unblinded (open-label) studies, (b) overall high risk of bias studies, (c) studies that did not use operationalized criteria for diagnosis, (d) studies with a duration shorter than 12 weeks, and (e) studies with specific patient characteristics (e.g., treatment-resistant, predominant negative symptoms).

#### Small study effects and reporting bias

We will explore the association between study size and effect size with a comparison-adjusted funnel plot [35–37]. Comparisons with more than 10 studies will be plotted in a contour-enhanced funnel plot. The possibility of reporting bias in the entire network will be assessed using the RoB-MEN framework [28].

#### Evaluating the confidence in estimates

The confidence in estimates of the main outcome will be evaluated with the framework Confidence in Network Meta-Analysis (CINeMA) [38], an adaptation of the Grading of Recommendations Assessment, Development, and Evaluation framework (GRADE) specifically developed for NMA. Within this framework, we will create tables with Summary of Confidence for the primary outcome “cognition composite score” and for the seven cognitive domains [38].

#### Statistical software

We will perform all analyses using the frequentist software R [39] (packages meta and netmeta [40, 41]). Network meta-regression will be performed in a Bayesian framework using self-programmed routines in JAGS [42].

## Discussion

This network meta-analysis will examine the effect of antipsychotic drugs on cognitive function in individuals with schizophrenia. Considering the large number of available substances, conventional pairwise meta-analysis is not capable of providing a sufficient overview. The analysis will benefit from maximal statistical power by combining direct and indirect comparisons in NMA, measuring the relative effects of the different antipsychotics on cognition. We will derive evidence-based hierarchies showing which antipsychotic has the largest effect in each cognitive domain.

Network meta-analysis currently presents the most advanced way to summarize evidence from multiple (in theory interchangeable) treatments, the meaningfulness of the obtained results is highly dependent on the quality of included studies. We expect high heterogeneity in the reporting of cognition measurements and a low percentage of studies that follow the actual MATRICS recommendations, given that we plan to include studies without restriction in terms of publication date. Including studies conducted over such a long timeframe, with differing methodologies and different patient populations such as children, individuals with treatment-resistant schizophrenia, or first-episode patients, poses a challenge to the assumption of transitivity needed for network meta-analysis. While we do consider it meaningful to include a variety of different study settings and participants, we will meticulously explore the sources of heterogeneity in the network. We will also be very strict in selecting the eligible test measures and matching them to the MATRICS domains to make the obtained values more comparable.

While addressing cognitive dysfunction in schizophrenia should not be restricted to antipsychotic drug choice, and cognitive remediation programs did show some effect on the cognitive symptoms of schizophrenia [43], we think it is of utmost importance to evaluate the differences between the antipsychotic substances in their effect on cognitive functioning—not only to inform drug choice, but also to identify possible links between the mechanism of action and effect on cognition of the various substances based on their receptor profiles.

## Supplementary Information

**Additional file 1.** PRISMA-P Checklist.

**Additional file 2.** Description of search strategy.

**Additional file 3.** Detailed search terms for the Cochrane Schizophrenia Trials Register.

## Data Availability

The datasets used and/or analyzed during the current study are available from the corresponding author upon reasonable request.

## Abbreviations

CINeMA: Confidence in Network Meta-Analysis
CSzG: Cochrane Schizophrenia Group
DSM: Diagnostic and Statistical Manual of Mental Disorders
GRADE: Grading of Recommendations, Assessment, Development, and Evaluation
ICD: International Classification of Diseases
LOCF: Last observation carried forward
MATRICS: Measurement and Treatment Research to Improve Cognition in Schizophrenia
MCCB: MATRICS Consensus Cognitive Battery
NMA: Network meta-analysis
PRISMA: Preferred Reporting Items of Systematic Reviews and Meta-Analyses for Systematic Review Protocols
PROSPERO: International Prospective Register of Systematic Reviews
RCT: Randomized controlled trial
RoB: Risk of bias
SD: Standard deviation
SE: Standard error
SIDE: Separating indirect evidence from direct evidence
SMD: Standardized mean difference

## References

[1] Vos T, Lim SS, Abbafati C, Abbas KM, Abbasi M, Abbasifard M (2020). Global burden of 369 diseases and injuries in 204 countries and territories, 1990–2019: a systematic analysis for the Global Burden of Disease Study 2019. Lancet.

[2] Harvey PD, Bosia M, Cavallaro R, Howes OD, Kahn RS, Leucht S (2022). Cognitive dysfunction in schizophrenia: an expert group paper on the current state of the art. Schizophr Res Cogn.

[3] Bowie CR, Harvey PD (2006). Cognitive deficits and functional outcome in schizophrenia. Neuropsychiatr Dis Treat.

[4] Dickson H, Hedges EP, Ma SY, Cullen AE, MacCabe JH, Kempton MJ (2020). Academic achievement and schizophrenia: a systematic meta-analysis. Psychol Med.

[5] Green MF (2016). Impact of cognitive and social cognitive impairment on functional outcomes in patients with schizophrenia. J Clin Psychiatry.

[6] Bouwmans C, de Sonneville C, Mulder CL, Hakkaart-van RL (2015). Employment and the associated impact on quality of life in people diagnosed with schizophrenia. Neuropsychiatr Dis Treat.

[7] Leucht S, Tardy M, Komossa K, Heres S, Kissling W, Salanti G (2012). Antipsychotic drugs versus placebo for relapse prevention in schizophrenia: a systematic review and meta-analysis. Lancet.

[8] Leucht S, Cipriani A, Spineli L, Mavridis D, Örey D, Richter F (2013). Comparative efficacy and tolerability of 15 antipsychotic drugs in schizophrenia: a multiple-treatments meta-analysis. Lancet.

[9] Huhn M, Nikolakopoulou A, Schneider-Thoma J, Krause M, Samara M, Peter N (2019). Comparative efficacy and tolerability of 32 oral antipsychotics for the acute treatment of adults with multi-episode schizophrenia: a systematic review and network meta-analysis. Lancet.

[10] Woodward ND, Purdon SE, Meltzer HY, Zald DH (2007). A meta-analysis of cognitive change with haloperidol in clinical trials of atypical antipsychotics: dose effects and comparison to practice effects. Schizophr Res.

[11] Nielsen RE, Levander S, KjaersdamTelléus G, Jensen SOW, Østergaard Christensen T, Leucht S (2015). Second-generation antipsychotic effect on cognition in patients with schizophrenia–a meta-analysis of randomized clinical trials. Acta Psychiatr Scand.

[12] Green MF, Nuechterlein KH, Kern RS, Baade LE, Fenton WS, Gold JM (2008). Functional co-primary measures for clinical trials in schizophrenia: results from the MATRICS Psychometric and Standardization Study. Am J Psychiatry.

[13] Kern RS, Nuechterlein KH, Green MF, Baade LE, Fenton WS, Gold JM (2008). The MATRICS Consensus Cognitive Battery, part 2: co-norming and standardization. Am J Psychiatry.

[14] Nuechterlein KH, Green MF, Kern RS, Baade LE, Barch DM, Cohen JD (2008). The MATRICS Consensus Cognitive Battery, part 1: test selection, reliability, and validity. Am J Psychiatry.

[15] Hutton B, Salanti G, Caldwell DM, Chaimani A, Schmid CH, Cameron C (2015). The PRISMA extension statement for reporting of systematic reviews incorporating network meta-analyses of health care interventions: checklist and explanations. Ann Intern Med.

[16] Feber L, Peter N. Antipsychotic drugs and their effects on cognitive function: a systematic review, pairwise and network meta-analysis. 2022. Available from: https://www.crd.york.ac.uk/prospero/display_record.php?RecordID=312483. Cited 2022 Aug 23. 10.1186/s13643-023-02213-5 PMC10037873 36959619

[17] Divine GW, Brown JT, Frazier LM (1992). The unit of analysis error in studies about physicians’ patient care behavior. J Gen Intern Med.

[18] Leucht S, Davis JM (2022). Which first-generation antipsychotics should be "repurposed" for the treatment of schizophrenia. Eur Arch Psychiatry Clin Neurosci.

[19] Gardner DM, Murphy AL, O’Donnell H, Centorrino F, Baldessarini RJ (2010). International consensus study of antipsychotic dosing. Am J Psychiatry.

[20] Nuechterlein KH, Barch DM, Gold JM, Goldberg TE, Green MF, Heaton RK (2004). Identification of separable cognitive factors in schizophrenia. Schizophr Res.

[21] Engel RR. TDB2Online-Dokumentation - Mehrfachstandardisierung auf unterschiedlichen Ebenen. 2022. Available from: https://psytest.psy.med.uni-muenchen.de/tdb2online/doku.php?id=dokumentation#mehrfachstandardisierung_auf_unterschiedlichen_ebenen. Cited 2022 Jul 26.

[22] COSMIN. COSMIN - improving the selection of outcome measurement instruments. 2022. Available from: https://www.cosmin.nl/. Cited 2023 Jan 11.

[23] Cochrane Schizophrenia - register of trials. Available from: https://schizophrenia.cochrane.org/register-trials. Cited 2022 Aug 2.

[24] List of Searches Resources. Available from: https://schizophrenia.cochrane.org/sites/schizophrenia.cochrane.org/files/public/uploads/List%20of%20Searches%20Resources.pdf. Cited 2022 Aug 2.

[25] Swiss Academic Software GmbH. Citavi (Version 6) [Software]. 2021. https://www.citavi.com/de.

[26] Higgins JPT, Thomas J, Chandler J, Cumpston M, Li T, Page MJ, Welch VA, editors. Cochrane Handbook for Systematic Reviews of Interventions. 2nd ed. Chichester: Wiley; 2019.

[27] Sterne JAC, Savović J, Page MJ, Elbers RG, Blencowe NS, Boutron I (2019). RoB 2: a revised tool for assessing risk of bias in randomised trials. BMJ.

[28] Chiocchia V, Nikolakopoulou A, Higgins JPT, Page MJ, Papakonstantinou T, Cipriani A (2021). ROB-MEN: a tool to assess risk of bias due to missing evidence in network meta-analysis. BMC Med.

[29] Turner RM, Davey J, Clarke MJ, Thompson SG, Higgins JP (2012). Predicting the extent of heterogeneity in meta-analysis, using empirical data from the Cochrane Database of Systematic Reviews. Int J Epidemiol.

[30] Rhodes KM, Turner RM, Higgins JPT (2016). Empirical evidence about inconsistency among studies in a pair-wise meta-analysis. Res Synth Methods.

[31] Salanti G, Higgins JPT, Ades AE, Ioannidis JPA (2008). Evaluation of networks of randomized trials. Stat Methods Med Res.

[32] Higgins JPT, Jackson D, Barrett JK, Lu G, Ades AE, White IR (2012). Consistency and inconsistency in network meta-analysis: concepts and models for multi-arm studies. Res Synth Methods.

[33] Dias S, Welton NJ, Caldwell DM, Ades AE (2010). Checking consistency in mixed treatment comparison meta-analysis. Stat Med.

[34] Rücker G, Schwarzer G (2015). Ranking treatments in frequentist network meta-analysis works without resampling methods. BMC Med Res Methodol.

[35] Chaimani A, Salanti G (2012). Using network meta-analysis to evaluate the existence of small-study effects in a network of interventions. Res Synth Methods.

[36] Leucht S, Leucht C, Huhn M, Chaimani A, Mavridis D, Helfer B (2017). Sixty years of placebo-controlled antipsychotic drug trials in acute schizophrenia: systematic review, bayesian meta-analysis, and meta-regression of efficacy predictors. Am J Psychiatry.

[37] Mavridis D, Sutton A, Cipriani A, Salanti G (2013). A fully Bayesian application of the Copas selection model for publication bias extended to network meta-analysis. Stat Med.

[38] Nikolakopoulou A, Higgins JPT, Papakonstantinou T, Chaimani A, Del Giovane C, Egger M (2020). CINeMA: an approach for assessing confidence in the results of a network meta-analysis. PLoS Med.

[39] R: a language and environment for statistical computing. Vienna: R Foundation for Statistical Computing; 2022. Available from: https://www.R-project.org/.

[40] Schwarzer G. Package ‘meta’: general package for meta-analysis. Available from: URL: https://cran.r-project.org/web/packages/meta/meta.pdf. Cited 2023 Feb 7.

[41] Rücker G, König J, Efthimiou O et al. Package ‘netmeta’: network meta-analysis using frequentist methods. Available from: https://cran.r-project.org/web/packages/netmeta/netmeta.pdf. Cited 2023 Feb 7.

[42] JAGS - Just Another Gibbs Sampler. 2022. Available from: https://mcmc-jags.sourceforge.io/. Cited 2023 Jan 11.

[43] Vita A, Barlati S, Ceraso A, Nibbio G, Ariu C, Deste G (2021). Effectiveness, core elements, and moderators of response of cognitive remediation for schizophrenia: a systematic review and meta-analysis of randomized clinical trials. JAMA Psychiat.

