# Supplementary material for: Antipsychotic drugs and their effects on cognitive function: protocol for a systematic review, pairwise, and network meta-analysis
Source: Syst Rev. 2023 Mar 24;12:54. doi: 10.1186/s13643-023-02213-5 (PMC10037873; doi:10.1186/s13643-023-02213-5)
Supplement: Supplementary file 2 — Additional file 2. Description of search strategy. [file 13643_2023_2213_MOESM2_ESM.docx]

**Additional file 2: Description of search strategy**

According to the methods outlined in the Cochrane handbook (1), the Cochrane Schizophrenia Group’s Information Specialist (FS) is collating this register from systematic searches of the different major resources and their updates (usually monthly).

- Cochrane Central Register of Controlled Trials (CENTRAL) in the Cochrane Library
- MEDLINE
- Embase
- Allied and Complementary Medicine (AMED)
- BIOSIS
- Cumulative Index to Nursing and Allied Health Literature (CINAHL)
- PsycINFO
- PubMed
- US National Institute of Health Ongoing Trials Register (ClinicalTrials.gov)
- World Health Organization International Clinical Trials Registry Platform ([www.who.int/ictrp](http://www.who.int/ictrp))
- ProQuest Dissertations and Theses A&I and its quarterly update

He is also adding results of handsearches and conference proceedings. The registry does not apply any restrictions in terms of language, date, document type or publication status (2).

The information specialist will then search the registry using the following terms:

(*Amisulpride* OR *Aripiprazole* OR *Asenapine* OR *Benperidol* OR *Brexpiprazole* OR *Cariprazine* OR *Chlorpromazine* OR *Clopenthixol* OR *Clozapine* OR *Flupentixol* OR *Fluphenazine* OR *Fluspirilene* OR *Haloperidol* OR *Iloperidone* OR *Levomepromazine* OR *Loxapine* OR *Lumateperone* OR *Lurasidone* OR *Molindone* OR *Olanzapine* OR *Paliperidone* OR *Penfluridol* OR *Perazine* OR *Perphenazine* OR *Pimozide* OR *Quetiapine* OR *Risperidone* OR *Sertindole* OR *Sulpiride* OR *Thioridazine* OR *Tiotixene* OR *Trifluoperazine* OR *Ziprasidone* OR *Zotepine* OR *Zuclopenthixol*) in Intervention Field of STUDY

As the register is study-based, a search by intervention will produce a list of all potentially relevant randomized controlled trials relevant to our research question. Having organised the reports already, and having them linked to the relevant topics (3) allows for more accurate search results that increase efficiency in the following steps of the systematic review process (4).
